# In the right order of brush strokes: a sketch of a software philosophy retrospective

**DOI:** 10.1186/2193-1801-3-186

**Published:** 2014-04-11

**Authors:** Evgeny Pyshkin

**Affiliations:** St. Petersburg State Polytechnical University, Institute of Computing and Control, Polytechnicheskaya ul., 21, 195021 St. Petersburg, Russia

**Keywords:** Software philosophy, Art, Software aesthetics, Liberal arts, Computer science education

## Abstract

This paper follows a discourse on software recognized as a product of art and human creativity progressing probably for as long as software exists. A retrospective view on computer science and software philosophy development is introduced. In so doing we discover parallels between software and various branches of human creative manifestations. Aesthetic properties and mutual dependency of the form and matter of art works are examined in their application to software programs. While exploring some philosophical and even artistic reflection on software we consider extended comprehension of technical sciences of programming and software engineering within the realm of liberal arts.

## Introduction

In about 60 years of its history software did a stressed way from an object of craft of professionals from highly selected club to the contemporary social scene. In recent years software manifested its pervasive nature considerably and became available to various groups of people.

More and more people become software writers, not only its users. To be an information-literate is one of the crucial demands for member of postindustrial society (Shapiro and Hughes 1996). In response, the nowadays software understanding leads us to the thesis that one of the most challenging aspects of current software development is its ability to transform and to change the world (DeMarco 2009). Attention to these aspects often overpasses pure engineering strategy to complete the project in time and within the resources limitations.

As noted in (Myers et al. 2011), “software we write today potentially touches millions of people, either enabling them to do their jobs effectively, or causing them untold frustration and costing them in the form of lost work or lost businesses”. Despite many formalized ways to represent data models, program structures, execution analysis and verification, and project organization were discovered, software engineering is still far from being an exact science. A psychologist Richie O’Bower noted that a programmer is rather not a mathematician but a philosopher and a linguist all in one: ability to program is not simply a kind of creative ability but the best one (O’Bower 1997). Sergei Arkhipenkov stated even more strongly: “software development is a kind of human activity which is *mistakenly* attributed to engineering” (Arkhipenkov 2012). But even if we accept this rather emotional idea, we still understand that it is impossible to say that writing software is either entirely art or entirely engineering. Truly, there is place for both components.

In software development there are disciplines where engineering prevails. It seems hard to provide good basis to estimate relative weights of art and engineering in software creation. In the latter days, in addition to traditional focus on problem solving, algorithms and data structures, hardware elements and architectures, design and development methodologies, new areas of computer science application are evolving. They include such areas as information retrieval, machine learning, social and ethical issues of the use of computers (and particularly in regards to software products).

Since computer science is connected with many interdisciplinary efforts, its specific boundaries become fuzzy (Walker and Kelemen 2009). Walker and Kelemen’s consideration goes even further when they concluded that computer science draws upon perspectives from many other disciplines. Hence, it has a symbiotic relationship with the liberal arts and therefore might be considered the ultimate of liberal arts disciplines. Interestingly, in medieval times the university curriculum differed remarkably from the today’s understanding of humanities: within the context of liberal arts students studied such disciplines as grammar, logic, arithmetic, geometry, music, rhetoric, and astronomy. Knuth noted that grammar, logic, and arithmetic are important components of computer science (Knuth 1974). Advancing with the consideration of computer science and especially software science as art, shouldn’t we stop with only three titles?

Since the first decades after emerging of computers and software related disciplines, both computer and liberal arts researchers attempted to explore some philosophy and artistic reflections on software engineering (Knuth 1974; Eden 2007; Wing 2006; Bond 2005). Since the appearance of software engineering in about late 60s, there were attempts to apply philosophical concepts to this discipline in order to reflect on engineers’ activities, but these attempts were rather limited.

Software engineering hasn’t been appropriately analyzed with highlighting a philosophical discourse on computer science. In addition to classical Knuth’s research (Knuth 2001) we can cite recent works in (Gruner 2011) and (Northover et al. 2008). Our current paper is another contribution to this yet unsystematic collection. However we concede eventual criticism on a bitty structure of this essay and evident lack of rational arguments and estimations, most of them being of quite suggestive nature.

## Good is beautiful, bad is ugly

The thesis about software aesthetic properties considered as products of programming creative nature, its complexity and its social significance isn’t novel. As far back as 1972 Andrey Ershov remarked: “in its creative nature programming goes a little further than most other professions, and comes to mathematics and writing” (Ershov 1972). In turn, Dijkstra remarked that it occurs that a computer program may fascinate us by its logical elegance, but appears often totally unfitted for the human perception (Dijkstra 1976). About a decade later Knuth mentioned that “computer programs are nice to write, and well-written computer programs are nice to read” (Knuth 1984). Knuth’s *Literate Programming* was then a novel transitional approach connecting (probably for the first time) a process of the source code creation with a process of the source code apprehension in terms of pleasantness that computer programs may effect. Later, the idea of writing programs as narratives found its reimplementation in other fields of software engineering, for example, in supporting acceptance test stories in behavior driven development.

However Arkhipenkov still complained in 2012: “I don’t need a language allowing writing good programs, I’m searching for a language making impossible to write bad programs.” Seems to be “crying out in the wilderness” (Matthew 3:1-3)? Indeed, if we only were blessed to have such a language in literature, we would read only outstanding novels.

It seems less realistic to wait for a software language guaranteeing good software: nothing changed over the years – excellent programs are written by excellent developers. Despite there is probably no reliable metric to measure how does the developers’ creativity affect their productivity and software product quality, there is intuitive expectation that it affects them strongly.

Here’s another example. Main points of the *Zen of Python* emphasize more surface impressions of software than its technical quality: beautiful is better than ugly not only in external appearance (Peters 2004). Wilson and Oram complain that university students are rarely taught how to see the software elegance, unlike to academic traditions in other creative fields like painting, plastic art or architecture (Oram and Wilson 2007). It is essential that architects study to know how to look at buildings, composers study to know how to learn from others’ music scores, but programmers mostly look at others’ works only to fix bugs. They don’t know how to see the code beauty. Unfortunately we often don’t have enough time for this.

Beautiful solutions aren’t obvious. Although a concept of (code) beauty are very subjective, there is a gut feeling: if a programmer is able to explain what makes the code beautiful, code beauty may be considered as one of properties allowing judging the software quality. The key question which is not answered here is what does beauty mean. This is a kind of term that we use without special definition. Here is another suggestive observation: often it is hard to find rationale for judging something beautiful or to convince that the code is beautiful. Unfortunately, it happens that people consider much easier to argue the inverse.

## The manner and matter in software compositions

In this article we follow our previous work focused on problems of programming teaching (Pyshkin 2011). With help of the philosophical categories of form and matter applied to software considered as a product of human creative ability, we believe that the form doesn’t simply clothe the content of the work and separates it from the outside being, just like if it was the case of literature, visual arts and even the case of music. The form rather connects the creation to the external world: the manner and matter being interpenetrating and mutually dependent (McElroy 1888)

In contrast to MacLennan’s consideration (MacLennan 2006), where the matter of software engineering is considered to be the hardware and the form is the software itself which organizes resources provided by the hardware into a dynamic purposeful process, we apply both categories to software. Our analogy is also in accord with Gruner’s mention from (Gruner 2011): “Like a poem, software has thus also aesthetic qualities (which are often forgotten in the literature on software ontology), such as *form* (even beauty in its form), legibility, etc.”

Hence, producing readable software is one of the essential abilities of a software engineer who, similar to a painter or a musician, programs not only the computer but also the act of reproduction of developer’s creation in the beholder’s mind.

Let us introduce a glance to Russian humanities. As Russian theologian and mathematician Pavel Florensky considered, paintings become works of art not at the moment of their creation, but at the moment when they are recognized by a recipient. Otherwise they aren’t more understandable than a music score (being a sort of two-dimensional graphics) before it sounds by instrumental or intellectual implementation. Nevertheless, composers say that an experienced musician is able to judge the music work’s value simply by viewing the music score graphics’ beauty or, on the contrary, it’s ugliness (Florensky 1991): they may be experienced to execute (and therefore to reproduce) the work mentally. Furthermore, a composer writing a symphony normally works with a music score which is an abstract representation of composer’s intentions. The adequacy of such a representation strongly depends on the author’s ability to map the notation to the sound mentally (Edmonds 2007). Remember the example of Beethoven who lost hearing in his later years.

Thus, not only the concepts underlie software development approaches, but also a sense of aesthetics that fast every enthusiastic developer has: “a sense of what is pleasant to perform and what is unpleasant to endure, what is beautiful to behold and what is intolerably ugly” (Bond 2005). It advances the Knuth’s note about programs’ beauty introduced as elegant statements of program’s tasks and *symphonic* (sic!) composition of its parts (Knuth 2001).

Since a source code is a textual form of a programming code (therefore these two types of codes are often supposed to be synonyms), the primary difficulty of code understanding is its interpretation as a textual artifact (Berry 2011). To a great extend, understanding software code is based on capabilities to deal with languages. Even for visual arts it is often hard to explain what makes the creation product beautiful. Zeki supposed that it is caused by the fact that the human brain’s visual system is much more developed than its language centers, since it has had much more time to evolve (Zeki and Nash 1999).

## Software art manifestations

As a matter of things, an idea to consider software as an art is quite recent, at least if we compare it to the genesis of software itself. But the question whether the criteria of other arts should be or may be applied to the domain of programming and software engineering, remains open.

We can recognize at least three types of software manifestations as an art. First, software as a media art, if the external appearance of software in form of interactive media is exploited at most. Second, “contestable”, or competitive art, if programmers compete in aesthetic appearance of programs written under some conditions that may be artificial. Contests of one-line programs could serve as a model. Third, and probably the most important for us, if we consider software internal implementation as an art, and discuss beauty attributes in the code itself.

Aesthetic satisfaction may come in different ways. Knuth mentioned that “pleasure is significantly enhanced when we accomplish something with limited tools” (Knuth 1974). I remember the epoch of so called programmable calculators with very restricted memory facilities. What a pleasure and proud it was, when I wrote a program solving a square equation and featuring to deal with complex roots. It had size of about 40 per cent less comparing to the “standard” solutions in published tutorials and used only high efficient stack memory (that could now be called as a processor cache) to manipulate with data instead of calculator registers which were accessible at much slower speed. It meant that I gained miraculously both execution time and memory space.

**Figure 1:**
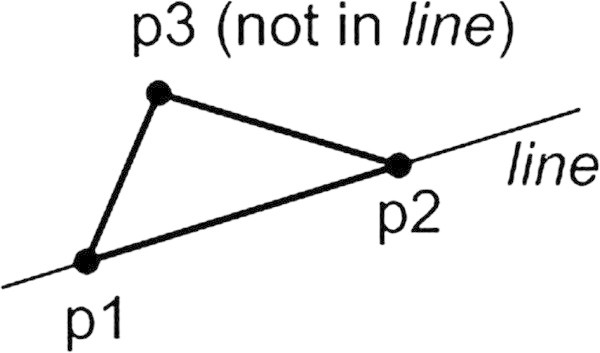
**Constructing a triangle.**



**Figure 2:**
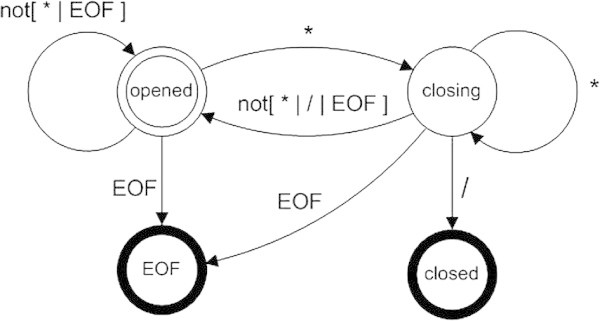
**Statechart for the closeComment() function.**

One more sample comes from an academic lab where I revised a student’s program dealing with constructing and processing triangles defined by the plain coordinates of its three angles. We converted the solution which primarily didn’t step over the bounds of six numeric values to a really nice construction defined in terms of geometry model presented in Figure 1 (do you still remember that geometry was one of the liberal arts in ancient universities?). Thus the solution has been shifted from the operational orientation to almost pure data model. The following fragment in Java provides some glimpse of that refactoring (see Listing 1):

Another sort of programming artistic manifestation is so called code poems. Inspired probably by the famous Perl poem by Hopkins (1995), the artist and engineer Ishac Bertran launched a project inviting people to write poetry in any programming language (Solon 2012). Indeed, many years ago, when I wrote a demo C functions to skip comments in style of ANSI C code, I felt vaguely that I wrote a sort of poem, in Bertran’s terms. Listing 2 represents one verse:

In contrast to the previous example dealing with “geometric types”, the above solution (for the process presented in Figure 2 as a state chart) is a kind of pure *control* structure (what makes it nice as I dare to say), the only data processing being the checking of the just scanned character.

So Knuth was right as he mentioned that even routine processes may sometimes be beautiful.

Another good example of such a routine process comes from the text processing domain. The task of parsing parenthesis-free expressions is one of traditional tasks used in the academic courses of programming. The problem of constructing an expression recursive parser is complex enough to explain elements of compiler theory, lexical and syntactic analysis, methods of parser construction, a concept of abstract syntax tree as well as the usage of polish notation to simplify expression calculation.

**Figure 3:**
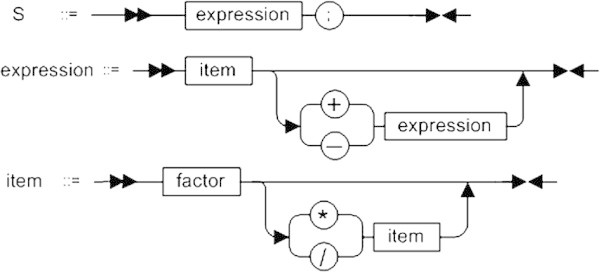
**Fragment of arithmetic expression grammar rules.**



At the same time an expression recursive parser is manageable to meet academic requirements. One could say that arithmetic expression recursive production rules illustrate a kind of ideal software requirements since the target program structure strongly relies on it. Isn’t it beautiful if the programming language implementation follows the grammar rules almost directly? The hierarchy of function or class method calls (Listing 3 represents a fragment of a parser class definition) may be considered as text based visualization of the production rules shown in Figure 3.

## Conclusion

Despite a computer program is primarily aimed to get some practical results of its execution, the object of a program isn’t effectively a heartless automaton performing sets of instructions represented in special form. The program is oriented to a human reader’s attention: we attempt to explain, what we would like to get from a computer and in which way. It gives us a newer comprehension of the Dijkstra’s note about programs that get their sense only during execution (Dahl and Hoare 1972). So, we may paraphrase on the earlier note: experienced software composers are equally able to judge the code value (i.e. code quality) by viewing code graphics’ beauty or, on the contrary, its ugliness.

There are software practices (extreme programming, for example) where code inspection is one of the essential production stages. Static analysis and unit testing are also examples of code recognition and rediscovering in the course of its verification. This leads us to one more interpretation of a Kent Beck’s famous maxim, “Hold on there – I never said that test-first was a testing technique. In fact, if I remember correctly, I explicitly stated that it wasn’t” (Beck 2001).

It’s hard to model adequately the human creative ability, but it’s possible to model the creative process itself, so to be able to recognize and to judge the artifacts being products of this process whether they are music compositions, pieces of painting, literary efforts or, in a case we are interested here in at most, software projects. Considering software as art changes our understanding of software and shifts the focus of the art from the object orientation to a broader system orientation (Edmonds 2007).

To conclude these rather scattered notes I’d like to cite two statements coming from the domains of fine arts and literature. I slightly revised them to produce some counterpoint to main ideas of this essay. Paul Klee stated that an eye follows the ways that were already managed inside the work^a^. Let’s note that these ways should be paved carefully and in the right order, just like in the Japanese calligraphy: *unless you write a kanji character in the right order of brush strokes, it would never look beautiful*! ^b^

## Endnotes

^a^ As quoted in George Perec’s *La Vie, mode d’emploi* (Perec 1978) (translated from French).

^b^ Paraphrased from Alex Kerr’s Lost Japan (Kerr 1996).
